# Volumetric thermometry

**DOI:** 10.1186/2050-5736-3-S1-O35

**Published:** 2015-06-30

**Authors:** Dennis Parker

**Affiliations:** 1 University of Utah, Salt Lake City, UT, United States

## Background/introduction

Many applications of thermal therapy would benefit from temperature distribution measurements with high spatial and temporal resolution that cover a large 3D volume. Although MRI can acquire 3D temperature measurements, it is not possible to obtain fully sampled 3D MRI measurements that cover the insonified field of view with sufficient spatial and temporal resolution for most thermal therapy procedures.

With multiple receiver coils, acquisition speed can be improved with undersampling and parallel image reconstruction methods. Constrained or model-based image reconstruction methods can allow increased undersampling. Depending on the anatomic region of interest, the effects of motion, chemical shift, and susceptibility need to be addressed. Non-Cartesian methods can reduce sensitivity to motion, but are less forgiving to off resonance effects.

Applications to the brain and the breast are considered.

## Methods

Brain: We have investigated several methods of k-space undersampling using temporally constrained reconstruction (TCR) and model-predictive filtering (MPF) methods. Volumetric MRTI was performed to test the ability to track heating throughout the volume, including the focus and critical points near the skull.

Breast: Although fat does not have frequency temperature dependence, it does have relaxation rates (T1, and T2) that change reproducibly with temperature. We have investigated hybrid MRTI sequences using dual flip angles to measure T1 simultaneously with PRF.

## Results and conclusions

Important observations include: 1) Volumetric MRTI measurements obtained during transcranial heating of plastic skull demonstrated temperature tracking at critical positions throughout the volume, including the focus and all areas near the skull. 2) Very large undersampling factors (up to R=12) were obtained and reconstructed with both TCR and MPF methods. 3) The TCR method is relatively accurate, but not real time and results in errors at times of rapid temperature transitions. 4) The MPF method yields volumetric MRTI measurements in real time to help guide treatment. Although MPF relies on estimates of tissue thermal properties and ultrasound SAR distribution, it is relatively insensitive to errors in these properties. Because MPF incorporates the transition times (on/off) of ultrasound power, rapid temperature transitions are more accurately represented. 5) With fully 3D measurements, band limited (zero-filled) interpolation can be performed in all three directions to decrease spacing between voxels and thereby decrease partial volume effects. 6) Reverse centric k-space trajectories, which can increase effective echo-time and increase temperature SNR, can result in a systematic T1-dependent error (downward bias) of measured temperature in narrowly focused heating patterns. 7) Sequential (non-centric) k-space trajectories can yield fast and accurate temperature measurements.

